# Job vacancies at the European Centre for Disease Prevention and Control

**DOI:** 10.2807/1560-7917.ES.2016.21.30.30303

**Published:** 2016-09-22

**Authors:** Eurosurveillance editorial team

**Affiliations:** 1 European Centre for Disease Prevention and Control (ECDC), Stockholm, Sweden

The European Centre for Disease Prevention and Control (ECDC) invites applications for the following positions:

• Head of Section Communication Support (PHC)

• Legal Advisor (RMC)

The deadline is 17 October 2016. For more information, visit the ECDC website: http://ecdc.europa.eu/en/aboutus/jobs/Pages/JobOpportunities.aspx

